# EXAMINING THE HEALTH EQUITY OF PEOPLE WITH DEMENTIA DURING THE COVID-19 PANDEMIC: FIRST INSIGHTS FROM A TWITTER STUDY

**DOI:** 10.1093/geroni/igac059.2810

**Published:** 2022-12-20

**Authors:** Juanita-Dawne Bacsu, Megan E O’Connell, Allison Cammer, Soheila Ahmadi, Corinne Berger, Mehrnoosh Azizi, Rory Gowda-Sookochoff, Karl Grewal

**Affiliations:** Thompson Rivers University, Kamloops, British Columbia, Canada; University of Saskatchewan, Saskatoon, Saskatchewan, Canada; University of Saskatchewan, Saskatoon, Saskatchewan, Canada; University of Saskatchewan, Saskatoon, Saskatchewan, Canada; University of Saskatchewan, Saskatoon, Saskatchewan, Canada; University of Saskatchewan, Saskatoon, Saskatchewan, Canada; University of Saskatchewan, Saskatoon, Saskatchewan, Canada; University of Saskatchewan, Saskatoon, Saskatchewan, Canada

## Abstract

The COVID-19 pandemic has deepened issues of health inequity and social injustice against people with dementia. Despite having one of the highest mortality rates, little research focuses on the COVID-19 impact of people with dementia. This presentation aims to: 1) explore the COVID-19 experiences and key factors of health inequity among people with dementia during the pandemic; and 2) identify actions to improve the health equity of people with dementia in the pandemic. We collected 6,243 relevant tweets using the Twint application in Python from September 8, 2020, to December 8, 2021. Tweets were divided among eleven coders and analyzed using thematic analysis. Analysis identified three primary themes: structural inequities (e.g., restricted access to health and support services, ageism, social isolation, vaccination barriers, and inadequate staffing in care facilities); frustration and despair due to loss (e.g., loss of cognitive abilities, loss of time with loved ones, and loss of life); and resiliency and hope for the future (e.g., lifting of restrictions and COVID-19 vaccine). There is an urgent need for policymakers to improve the health equity of people with dementia in the pandemic. Tackling COVID-19 inequities requires revisiting infection control policies to improve access to health and support services, recognizing the essential role of family care partners, and providing resources to help support people with dementia during the pandemic. Moreover, it is essential that COVID-19 policy responses are informed by evidence-informed research and authentic partnerships that embrace the insight and lived experiences of people with dementia.

